# Found and Lost: The Fates of Horizontally Acquired Genes in Arthropod-Symbiotic *Spiroplasma*

**DOI:** 10.1093/gbe/evv160

**Published:** 2015-08-08

**Authors:** Wen-Sui Lo, Gail E. Gasparich, Chih-Horng Kuo

**Affiliations:** ^1^Institute of Plant and Microbial Biology, Academia Sinica, Taipei, Taiwan; ^2^Molecular and Biological Agricultural Sciences Program, Taiwan International Graduate Program, NationalChung Hsing University and Academia Sinica, Taipei, Taiwan; ^3^Graduate Institute of Biotechnology, National Chung Hsing University, Taichung, Taiwan; ^4^Department of Biological Sciences, Towson University; ^5^Biotechnology Center, National Chung Hsing University, Taichung, Taiwan

**Keywords:** Mollicutes, *Spiroplasma*, genome degradation, pseudogene, horizontal gene transfer (HGT), mobile genetic element (MGE)

## Abstract

Horizontal gene transfer (HGT) is an important mechanism that contributed to biological diversity, particularly in bacteria. Through acquisition of novel genes, the recipient cell may change its ecological preference and the process could promote speciation. In this study, we determined the complete genome sequence of two *Spiroplasma* species for comparative analyses and inferred the putative gene gains and losses. Although most *Spiroplasma* species are symbionts of terrestrial insects, *Spiroplasma eriocheiris* has evolved to be a lethal pathogen of freshwater crustaceans. We found that approximately 7% of the genes in this genome may have originated from HGT and these genes expanded the metabolic capacity of this organism. Through comparison with the closely related *Spiroplasma atrichopogonis*, as well as other more divergent lineages, our results indicated that these HGT events could be traced back to the most recent common ancestor of these two species. However, most of these horizontally acquired genes have been pseudogenized in *S. atrichopogonis*, suggesting that they did not contribute to the fitness of this lineage that maintained the association with terrestrial insects. Thus, accumulation of small deletions that disrupted these foreign genes was not countered by natural selection. On the other hand, the long-term survival of these horizontally acquired genes in the *S. eriocheiris* genome hinted that they might play a role in the ecological shift of this species. Finally, the implications of these findings and the conflicts among gene content, 16S rRNA gene sequencing, and serological typing, are discussed in light of defining bacterial species.

## Introduction

Horizontal gene transfer (HGT), a process in which the recipient cell acquires genetic information from sources other than its parental cell, is important for generating biological diversity in bacteria (Ochman et al. 2000; Gogarten et al. 2002; Gogarten and Townsend 2005; Lerat et al. 2005; Wiedenbeck and Cohan 2011). Based on large-scale analyses across diverse lineages, at least approximately 75–81% of the gene families in bacterial genomes have been involved in HGT (Dagan et al. 2008; Kloesges et al. 2011). Although many of the horizontally acquired genes may not contribute to the fitness of their new host genomes (Kuo and Ochman 2009a) and are expected to be removed by the mutational bias toward deletions commonly observed in bacteria (Mira et al. 2001; Kuo and Ochman 2009b, 2010), in some cases these new genes could contribute to adaptation, promote ecological divergence, and eventually lead to speciation (Pál et al. 2005; Luo et al. 2011; Zhu et al. 2014).

The occurrence of HGT is often facilitated by the overlap in ecological niches between the donor and the recipient cells (Hooper et al. 2009; Halary et al. 2010). For example, mobile genetic elements (MGEs) such as transposons or phages have been shown to facilitate the genetic exchanges among bacteria forming biofilm together (Madsen et al. 2012) or infecting the same eukaryotic host (Chafee et al. 2010). Based on the microbial diversity of the ecological niches occupied, HGT is expected to occur frequently among free-living bacteria that live in complex communities (Ochman and Davalos 2006). In contrast, HGT is rare in obligate intracellular symbionts because of their isolation from other bacteria and their losses of genes involved in DNA uptake and recombination (Silva et al. 2003). Bacterial lineages that have adapted to a symbiotic lifestyle recently or have maintained a facultative relationship with their eukaryotic hosts are expected to experience intermediate levels of HGT. However, the exact contribution of HGT in the evolution of facultative symbionts remains to be better characterized.

**Fig. 1.—:**
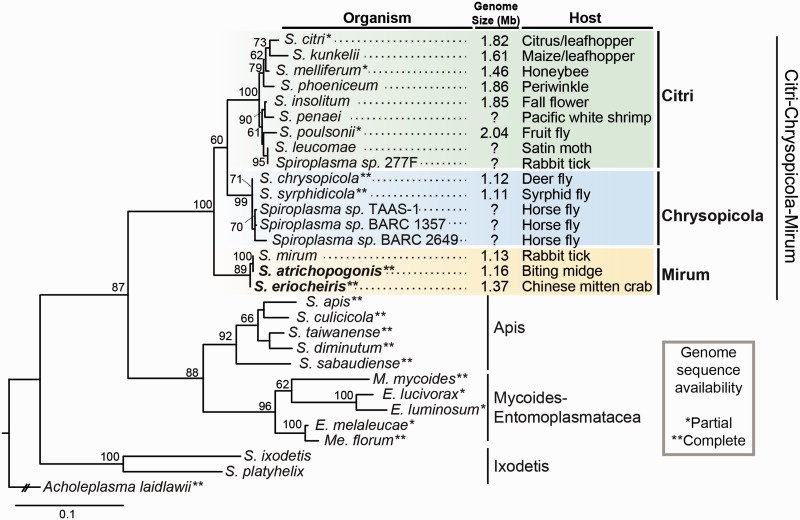
Molecular phylogeny of *Spiroplasma*. The maximum-likelihood phylogeny is based on the 16S rRNA gene. Bootstrap values ≥60% are labeled above the internal branches. The taxon sampling is focused on the Citri–Chrysopicola–Mirum clade within the genus; the isolation source is labeled after the strain name. The two strains analyzed in this study are highlighted in bold. The genus *Spiroplasma* is a paraphyletic group; lineages belonging to the Mycoides–Entomoplasmatacea clade include species assigned to *Mycoplasma* (*M*.), *Mesoplasma* (*Me*.), and *Entomoplasma* (*E*.). The accession numbers are provided in supplementary table S1, Supplementary Material.

The genus *Spiroplasma* contains diverse species that are mostly facultative symbionts of terrestrial insects (fig. 1). Their lifestyles range from harmless commensals, beneficial symbionts, to pathogens with varying levels of host-dependence (Whitcomb 1981; Gasparich et al. 2004; Regassa and Gasparich 2006; Gasparich 2010). Thus, comparisons among *Spiroplasma* lineages could improve our understanding of the transition between ecological niches and shed light on the evolution of symbiosis (Anbutsu and Fukatsu 2011). Previous comparative genomic analyses within this genus have revealed several interesting points related to HGT. First, the species belonging to the Citri clade have experienced extensive viral invasion in their genomes, which facilitated genome rearrangements and HGT (Carle et al. 2010; Alexeev et al. 2012; Lo, Chen, et al. 2013; Paredes et al. 2015). In contrast, species belonging to the sister Chrysopicola clade lack such integration of foreign genetic materials, presumably due to the presence of CRISPR and restriction modification systems (Ku et al. 2013). For species belonging to the more divergent Apis clade, no notable MGE has been reported but HGT appears to have shaped the evolution of putative virulence factors in several mosquito symbionts (Lo, Ku, et al. 2013; Chang et al. 2014).

In this study, we further expanded the taxon sampling of genome analyses in *Spiroplasma*, specifically focusing on the Mirum clade that is sister to the Citri–Chrysopicola clade (fig. 1). One species in this clade, *Spiroplasma eriocheiris* (Wang et al. 2011), is a lethal pathogen of several freshwater crustaceans, including the Chinese mitten crab (*Eriocheir sinensis*), red swamp crayfish (*Procambarus clarkia*), and Pacific white shrimp (*Penaeus vannamei*) (Bi et al. 2008). To investigate the genome evolution events that may be associated with this switch of hosts from terrestrial insects to aquatic crustaceans, we conducted comparative analyses between *S. eriocheiris *and a closely related species *Spiroplasma atrichopogonis *(Koerber et al. 2005), which is associated with biting midges (*Atrichopogon geminus* and *Atrichopogon levis*), and focused on the inference of putative gene gains and losses.

## Materials and Methods

### Bacterial Strains

The type strain *S. atrichopogonis* ATCC BAA-520^T^ (=GNAT3597^T^) (Koerber et al. 2005) was acquired from the American Type Culture Collection, and *S. eriocheiris* DSM 21848^T^ (=TDA-040725-5^T^) (Wang et al. 2011) was acquired from the German Collection of Microorganisms and Cell Cultures. The freeze-dried samples were rehydrated by adding 5 ml of liquid M1D medium (Whitcomb et al. 1982), titrated by serial dilution, and incubated at 30 °C without shaking until the medium turned yellow. The minimum concentration that showed bacteria growth was then transferred to fresh medium for propagation, followed by DNA extraction using the Wizard Genomic DNA Purification Kit (Promega, USA). For each DNA sample, we amplified the 16S rRNA gene using the primer pair 8F (5′-agagtttgatcctggctcag-3′) (Turner et al. 1999) and 1492R (5′-ggttaccttgttacgactt-3′) (Ochman et al. 2010) for Sanger sequencing to confirm the sample identity.

The 16S sequence of our *S. eriocheiris* DSM 21848^T^ sample matched the type strain record (GenBank accession number DQ917753). The 16S sequence of our *S. atrichopogonis* ATCC BAA-520^T^ sample (GenBank accession number KR349131) matched that from the original isolate *S. atrichopogonis* GNAT3597^T^ maintained in Dr Gail Gasparich’s laboratory at Towson University in the United States (GenBank accession number KR349130) but not the second duplicate deposited in Japan (*S. atrichopogonis* NBRC 100390^T^; GenBank accession number AB681165). For this reason, we believe that the *S. atrichopogonis* ATCC BAA-520^T^ sample reported in this study represents the type strain of this species. On the other hand, the 16S sequence of NBRC 100390 indicates that it is not a duplicate of the type strain of *S. atrichopogonis* as reported (Koerber et al. 2005). Rather, the strain NBRC 100390 appeared to be closely related to *Spiroplasma insolitum* in the Citri clade of this genus. The discrepancy has been reported to and acknowledged by the NBRC.

### Genome Sequencing, Assembly, and Annotation

The procedures for genome sequencing, assembly, and annotation were based on those described in our previous studies (Chung et al. 2013; Lo, Chen, et al. 2013; Lo, Ku, et al. 2013). For each strain, one paired-end library was prepared and sequenced on the MiSeq (Illumina, USA) platform. The average insert size was approximately 550 bp and the read length was 301 bp from each end. Approximately 0.8 and 0.5 Gb of raw reads were obtained for *S. atrichopogonis* and *S. eriocheiris*, respectively. The initial de novo assembly was generated using VELVET v1.2.07 (Zerbino and Birney 2008) and iteratively improved using IMAGE (Swain et al. 2012). At the end of each iteration, the raw reads were mapped to the assembly using BWA v0.7.4 (Li and Durbin 2009), programmatically checked using the MPILEUP program in SAMTOOLS package v0.1.18 (Li et al. 2009), and visualized using IGV v2.3.51 (Robinson et al. 2011). The process was repeated until all gaps were closed for each circular chromosome.

The complete genome sequences were processed using RNAmmer (Lagesen et al. 2007), tRNAscan-SE (Lowe and Eddy 1997), and PRODIGAL (Hyatt et al. 2010) for gene prediction. The draft annotation of protein-coding genes was based on the homologous genes in other *Spiroplasma* genomes (table 1) as identified by OrthoMCL (Li et al. 2003) with a BLASTP (Camacho et al. 2009) *e*-value cutoff of 1 × 10^−^^15^. Subsequently, SignalP v4.0 (Petersen et al. 2011), TMHMM v2.0 (Krogh et al. 2001), and the Conserved Domain Database (Marchler-Bauer et al. 2013) were used to provide additional information on signal peptides, transmembrane domains, and conserved protein domains, respectively. Finally, BLASTP searches against the National Center for Biotechnology Information (NCBI) nonredundant protein database (Benson et al. 2015) and KAAS (Moriya et al. 2007) searches against the KEGG database (Kanehisa and Goto 2000; Kanehisa et al. 2010) were performed to facilitate manual curation. To identify pseudogenes, we examined genes with a truncated open reading frame that is less than 70% of the median length of its homologous genes in other *Spiroplasma* genomes (table 1). These candidates, along with their neighboring open reading frames, were then checked for deletions, frameshift mutations, or premature stop codons during the manual curation. The final annotated chromosomes were plotted using CIRCOS (Krzywinski et al. 2009) to show gene locations, GC-skew, and GC content.

**Table 1:** Genome Features of Selected *Spiroplasma* Strains

Genome	Chromosomal Contigs	Combined Size (bp)	G+C Content (%)	Coding Density (%)	Protein-Coding Genes	Length Distribution (Q1/Q2/Q3) (amino acids)	Plectroviral Genes	Annotated Pseudogenes	rRNA Operon	tRNA Genes	Plasmids
*Spiroplasma citri *GII3-3X	AM285301–AM285339	1,525,756	25.9	80.2	1,222	83/149/286	375	0	1	32	7
*Spiroplasma melliferum *IPMB4A	AMGI01000001–AMGI01000024	1,098,846	27.5	85.1	932	176/280/440	11	12	1	32	0
*Spiroplasma melliferum *KC3	AGBZ02000001–AGBZ02000004	1,260,174	27.0	84.4	1,197	141/248/403	6	28	1	31	2
*Spiroplasma poulsonii *MSRO	JTLV01000001–JTLV01000010	1,757,846	26.5	76.6	2,129	83/150/278	94	82	1	31	2
*Spiroplasma chrysopicola* DF-1	CP005077	1,123,322	28.8	89.0	1,009	171/278/425	0	6	1	33	0
*Spiroplasma syrphidicola *EA-1	CP005078	1,107,344	29.2	90.4	1,006	176/279/425	0	3	1	32	0
*Spiroplasma atrichopogonis *GNAT3597	CP011855	1,160,484	29.3	71.5	996	108/214/362	0	158	1	33	0
*Spiroplasma eriocheiris *TDA-040725-5	CP011856	1,365,714	29.8	86.0	1,180	168/278/423	0	30	1	32	0

### Phylogenetic and Comparative Analyses

For molecular phylogenetic inference, the putative homologs of each gene were identified by Basic Local Alignment Search Tool (BLAST) searches against the NCBI nonredundant nucleotide (for 16S rRNA gene) or protein (for selected protein-coding genes) database using the *S. eriocheiris* sequence as the query. The BLAST *e*-value cutoff was set at 1 × 10^−^^15^ and the hits were manually selected for optimal taxon sampling. The accession numbers are provided in supplementary table S1, Supplementary Material online. The multiple sequence alignment was performed using MUSCLE v3.8 (Edgar 2004). The maximum-likelihood phylogenies were inferred using PhyML v3.0 (Guindon and Gascuel 2003). The proportion of invariable sites and the gamma distribution parameter were estimated from the data set and the number of substitute rate categories was set to 4. Bootstrap supports were estimated based on 1,000 replicates generated by the SEQBOOT program of PHYLIP v3.69 (Felsenstein 1989).

For gene content comparison, the genomes belonging to the Citri–Chrysopicola clade (table 1) were merged into one pan-genome prior to homologous gene identification with *S. atrichopogonis* and *S. eriocheiris *using OrthoMCL. The inference of metabolic pathways was based on the available annotation and the KEGG pathway tool (Kanehisa and Goto 2000; Kanehisa et al. 2010). The examination of syntenic regions was performed manually based on BLASTP searches among the genomes being compared.

## Results

### Phylogenetic Placement of *S. atrichopogonis* and *S. eriocheiris*

When *S. atrichopogonis* was first described as a novel species in 2005 (Koerber et al. 2005), the type strain GNAT3597^T^ was found to be serologically distinct from other representative *Spiroplasma* strains of established groups. The only notable clue was that it had a slight reaction to the antiserum of *Spiroplasma mirum* SMCA^T^ in the deformation test at 160-fold dilution. Because group placement requires reciprocal positive deformation results at ≥320-fold dilution (Brown 2010) and no molecular phylogenetic inference was performed at that time, the phylogenetic affiliation of *S. atrichopogonis* has remained unknown. With the availability of its full 16S rRNA gene sequence from our genome sequencing effort, we inferred the phylogenetic placement of *S. atrichopogonis* (fig. 1). The result indicated that *S. atrichopogonis* GNAT3597^T^ is sister to *S. mirum* SMCA^T^ and the two strains share 100% sequence identity in their 16S rRNA genes.

The 16S rRNA gene of *S. eriocheiris* TDA-040725-5^T^ differs from *S. atrichopogonis*/*S. mirum* by 4 bp (1,506/1,510 = 99.7% identity). Based on the initial characterization of *S. eriocheiris* (Wang et al. 2011) and the 16S rRNA phylogeny of this study (fig. 1), *S. eriocheiris* was confidently placed in the Mirum clade within the genus *Spiroplasma*. It is interesting to note that despite the high 16S rRNA sequence identity, *S. eriocheiris* and *S. mirum* were assigned to different serological groups based on the deformation test as well. In the reciprocal test, *S. eriocheiris* showed a positive deformation result at 80-fold diluted *S. mirum* antiserum and *S. mirum* showed a positive deformation result at 160-fold diluted *S. eriocheiris* antiserum (Wang et al. 2011). As these cross-reactions did not reach the 320-fold dilution threshold (Brown 2010), *S. eriocheiris* was placed in a novel serological group XLIII that is distinct from the *S. mirum* (group V).

### Genome Features of *S. atrichopogonis* and *S. eriocheiris*

**Fig. 2.—:**
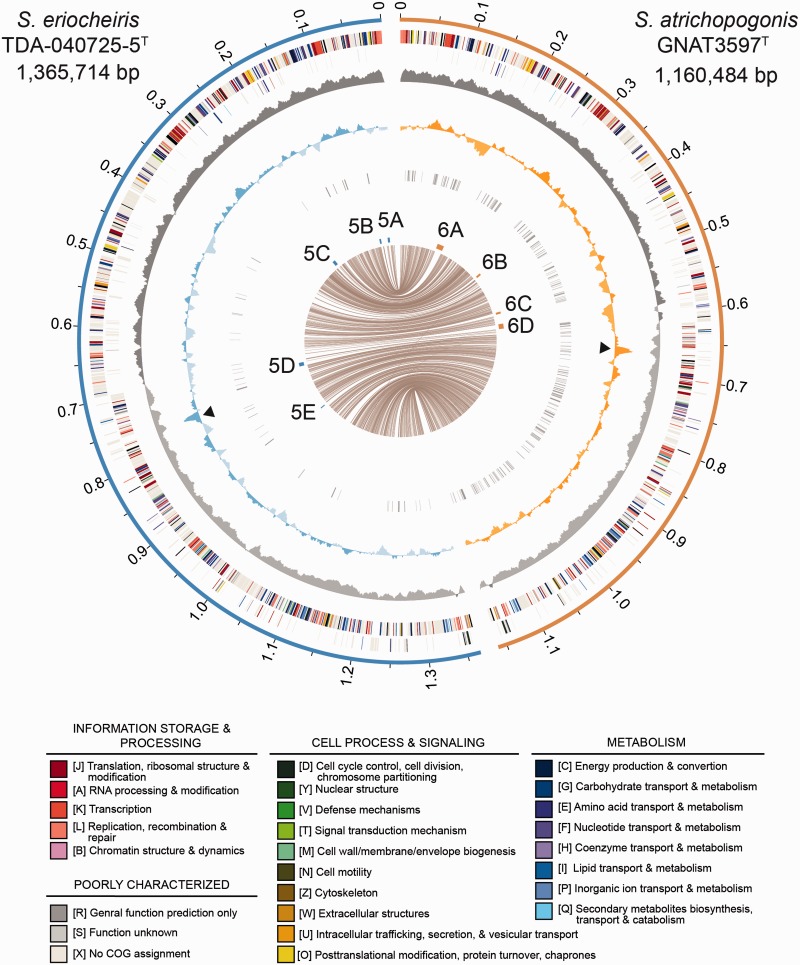
Chromosomal organization of *S. atrichopogonis and S. eriocheiris*. Concentric circles from the outside in (1) scale marks (unit: Mb), (2 and 3) protein-coding genes on the forward and reverse strand, respectively (color-coded by the functional categories), (4) GC skew (positive, dark gray; negative, light gray), (5) GC content (above average, dark shade; below average, light shade; rRNA operons are labeled by black triangles), (6) intergenic regions greater than 600 bp and annotated pseudogenes, (7) genomic regions shown in figures 5 and 6, and (8) lines linking single-copy orthologs shared between the two genomes.

The genome features of *S. atrichopogonis *and *S. eriocheiris *were summarized in table 1. Both genomes contain a single circular chromosome with a GC content of approximately 30%, which is comparable to those found in the Chrysopicola clade and higher than the Citri clade. *Spiroplasma atrichopogonis* has a smaller chromosome than *S. eriocheiris* does (1,160,484 and 1,365,714 bp, respectively) and fewer protein-coding sequences (CDS; 996 and 1,180, respectively). These differences are mainly explained by accumulation of many small-scale deletions in *S. atrichopogonis*. The *S. atrichopogonis* genome harbors 158 annotated pseudogenes (154 of which have intact orthologs in *S. eriocheiris*) and 83 long intergenic regions (i.e., >600 bp; possibly contain highly degraded pseudogenes that cannot be annotated) dispersed throughout its chromosome (fig. 2), which resulted in a coding density of only 71.5%. This coding density is the lowest one among the 13 *Spiroplasma* genomes reported to date (Carle et al. 2010; Alexeev et al. 2012; Lo, Chen et al. 2013; Ku et al. 2013; Lo, Ku et al. 2013; Ku et al. 2014; Chang et al. 2014; Paredes et al. 2015). In contrast, the *S. eriocheiris* genome contains only 30 pseudogenes and 19 long intergenic regions, which resulted in a coding density of 86.0% (table 1). Furthermore, comparison of 756 single-copy genes conserved between these two strains revealed that the *S. atrichopogonis* CDS are approximately 6.0% shorter than their orthologs in *S. eriocheiris*, which accounts for approximately 44 kb of the difference in their chromosome sizes.

Despite the aforementioned differences, the correspondence of single-copy orthologs between these two strains showed a high level of conserved synteny (fig. 2). The patterns of GC skew further supported that no major chromosomal rearrangement had occurred recently; only one inconsistency was found near the replication terminus of *S. atrichopogonis* (at ∼0.6 Mb), which involved the insertion of a putative transposon. This high level of genome stability is similar to those found in the Chrysopicola clade (Ku et al. 2013). The extensive genome rearrangements linked to plectroviral invasion as those observed in the Citri clade lineages (Lo, Chen, et al. 2013; Ku et al. 2013) have not occurred in these two newly sequenced strains belonging to the Mirum clade. Taken together, the 16S rRNA phylogeny (fig. 1) and the conservation in chromosomal organization (Belda et al. 2005) both support that the divergence between these two strains had occurred only recently.

### Comparison of Gene Content and Substrate Utilization Strategies

**Fig. 3.—:**
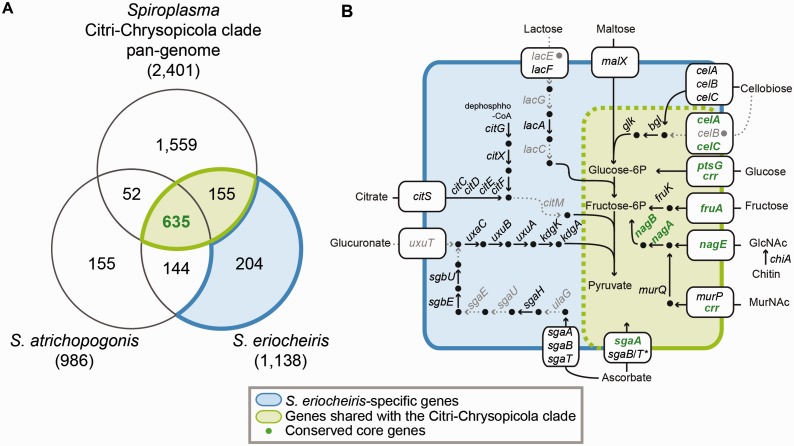
Gene content analysis. (*A*) A Venn diagram showing the distribution of homologous gene clusters among *S. atrichopogonis*, *S. eriocheiris*, and the pan-genome of those Citri–Chrysopicola clade strains listed in table 1. The *S. eriocheiris *homologs are divided into strain-specific genes (blue; 204 clusters) and those shared with the Citri–Chrysopicola clade (green; including 635 clusters with intact homologs in *S. atrichopogonis* and 155 without, all of which are putatively ancestral to the Citri–Chrysopicola–Mirum clade). Other than the 635 genes clusters shared by all, most of the *S. atrichopogonis* genes (including the 144 clusters shared with *S. eriocheiris* but not the Citri-Chrysopicola clade) are hypothetical proteins and were excluded from the metabolic inference. The complete lists of genes are provided in supplementary table S2, Supplementary Material online. (*B*) Selected metabolic pathways of *S. eriocheiris*. The color scheme is based on that used in panel (*A*); pathway in dashed lines and gene names in gray color indicate that the corresponding genes are absent or have been pseudogenized (labeled by filled gray circles).

Previous studies suggested that the pathogenicity and adaptation to hosts in *Spiroplasma* are often associated with their substrate utilization capabilities (Gaurivaud et al. 2000; André et al. 2005; Lo, Chen, et al. 2013; Lo, Ku, et al. 2013; Chang et al. 2014). To infer the gene content evolution, we compared *S. atrichopogonis *and *S. eriocheiris* with the pan-genome of *Spiroplasma* strains belonging to the Citri–Chrysopicola clade (fig. 3 and supplementary table S2, Supplementary Material online). Because of the extensive gene losses in *S. atrichopogonis*, we considered the 155 gene clusters shared between *S. eriocheiris *and the Citri–Chrysopicola clade, together with the 635 clusters shared by all, as ancestral to the Citri–Chrysopicola–Mirum clade (fig. 3*A*).

The metabolic inference suggests that the genes involved in the utilization of cellobiose, glucose, fructose, chitin, *N*-acetylglucosamine (GlcNAc), *N*-acetylmuramic acid (MurNAc), and ascorbate are likely to be ancestral to the Citri–Chrysopicola–Mirum clade (fig. 3*B*). Because these genes are present in the more distantly related species belonging to the Apis clade (Lo, Ku, et al. 2013; Chang et al. 2014), it is reasonable to assume that the most recent common ancestor (MRCA) of *Spiroplasma* could utilize these substrates as well. However, some of these substrates may not be universally important for the arthropod-symbiotic lifestyle of these bacteria. For example, genes related to the utilization of cellobiose (*celB*, *bgl*, and *glk*), fructose (*fruK*), chitin (*chiA*), MurNAc (*murP* and *murQ*), and ascorbate (*sgaB/T*) all have been lost in *S. atrichopogonis *(fig. 3*B*), as well as in some other *Spiroplasma* species (Lo, Chen, et al. 2013; Lo, Ku, et al. 2013; Chang et al. 2014). In contrast, genes involved in the utilization of glucose and GlcNAc are well conserved among the *Spiroplasma* lineages with complete genome sequences available (Lo, Ku, et al. 2013; Chang et al. 2014). This finding suggests that these substrates may be essential carbon sources for these bacteria and provides useful clues for future development of culture media for *Spiroplasma*.

In comparison to the Citri–Chrysopicola clade, genes for oligopeptide ABC transporter (*oppA*, *oppB*, *oppC*, *oppD*, and *oppF*), thiol peroxidase (*tpx*), ribonuclease P (*rnpA*), and uracil permease (*pyrP*) are absent in both *S. atrichopogonis *and *S. eriocheiris*. Because these genes are present in the Apis clade (Lo, Ku, et al. 2013; Ku et al. 2014; Chang et al. 2014), these gene losses were likely to have occurred in the MRCA of the Mirum clade. Furthermore, the type II CRISPR system (including *cas1* and *cas2* for foreign DNA recognition and *cas9* for endonuclease activity on invading DNA) appeared to have been degraded in both of these Mirum clade species. In *S. eriocheiris*, although *cas2* and *cas9* are still intact, *cas1* is truncated. In the more degenerated genome of *S. atrichopogonis*, only *cas2* is still intact, whereas *cas9* is truncated and *cas1* is absent.

One interesting observation of our gene content analysis is that *S. eriocheiris* has 214 genes (assigned to 204 homologous gene clusters, see fig. 3*A*) without identifiable homologs in other *Spiroplasma* genomes. Several of these genes appeared to have been integrated in the metabolic network of this bacterium, such as maltose transporter (*malX*), citrate uptake and utilization (*citS*, *citC*, *citD*, *citE*, and *citF*), glucuronate metabolism (*uxaC*, *uxuB*, *uxuA*, *kdgK*, and *kdgA*), as well as a second set of genes for the cellobiose (*celA*, *celB*, and *celC*) and ascorbate (*sgaA*, *sgaB*, and *sgaT*) transporters (fig. 3*B*). The last case is of particular interest because the *sgaB/T* genes are fused in the first set (green in fig. 3; as found in other *Spiroplasma*) while remaining as two separate genes in the second set (blue in fig. 3; as found in Firmicutes).

In contrast to *S. eriocheiris*, where many of the strain-specific genes were found to be involved in metabolism, almost all of the *S. atrichopogonis*-specific genes were annotated as hypothetical proteins with unknown functions. Examination of the gene content revealed that several genes involved in DNA repair (*recO*, *recR*, *recU*, and *ruvB*), as well as DNA polymerase IV (*dinB*), are either pseudogenized or absent in *S. atrichopogonis*. These gene losses are expected to increase the mutation accumulation rate. Consistent with this expectation, the protein phylogeny based on the concatenation of 507 single-copy genes shared by all *Spiroplasma* genomes listed in table 1 shows that *S. atrichopogonis *has a longer terminal branch than *S. eriocheiris* does (0.036 and 0.016, respectively). This higher mutation accumulation rate, together with the much shorter length of strain-specific genes in *S. atrichopogonis* (average = 265 bp; Q1/Q2/Q3 = 162/228/327 bp) compared with *S. eriocheiris* (average = 830 bp; Q1/Q2/Q3 = 329/663/1,063 bp), suggests that a large proportion of these *S. atrichopogonis*-specific genes may be fragments of unrecognizable pseudogenes.

### HGT in *S. eriocheiris*

**Fig. 4.—:**
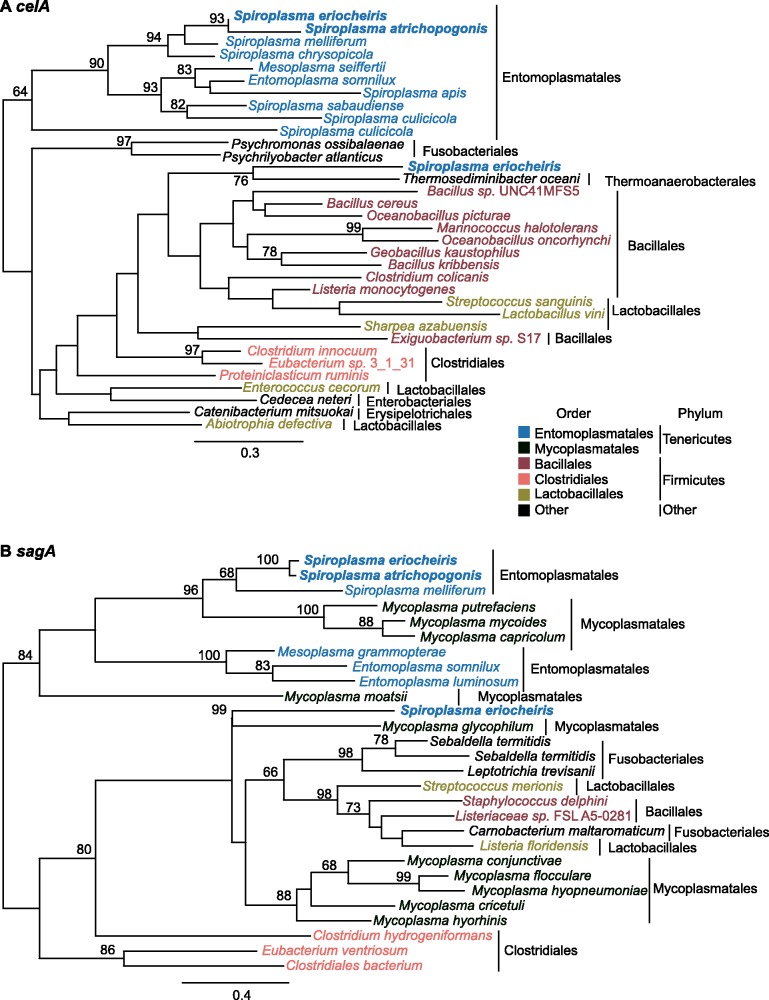
Molecular phylogenies of *celA* and *sgaA*. *Spiroplasma eriocheiris* has two sets of cellobiose (*celA*; panel *A*) and ascorbate (*sgaA*; panel *B*) transporter genes. Gene phylogenies demonstrate that one copy clustered with homologs from other Tenericutes lineages (suggesting vertical heritance), whereas the other clustered with homologs from Firmicutes (suggesting possible horizontal transfer). The accession numbers are provided in supplementary table S1, Supplementary Material online.

The gene content analysis, as well as several unexpected patterns found during the annotation process (e.g., ∼7% of the *S. eriocheiris* genes have the best BLASTP hit outside of the genus), led to the suspicion that HGT may be an important factor that contributed to the genome evolution of this strain. The molecular phylogenies of *celA* (fig. 4*A*) and *sagA* (fig. 4*B*) provided two examples to illustrate this point. As noted in the gene content analysis (fig. 3*B*), two copies were found for these transporter genes. In both cases, one copy clusters with homologs from other Entomoplasmatales/Mycoplasmatales lineages in the phylum Tenericutes, whereas the other clusters with homologs from the more distantly related lineages in the phylum Firmicutes. These results suggested that the two copies of *celA*/*sagA* are not paralogs that arose from within-genome gene duplications. Rather, the second copy was likely to have been acquired from HGT events.

**Fig. 5.—:**
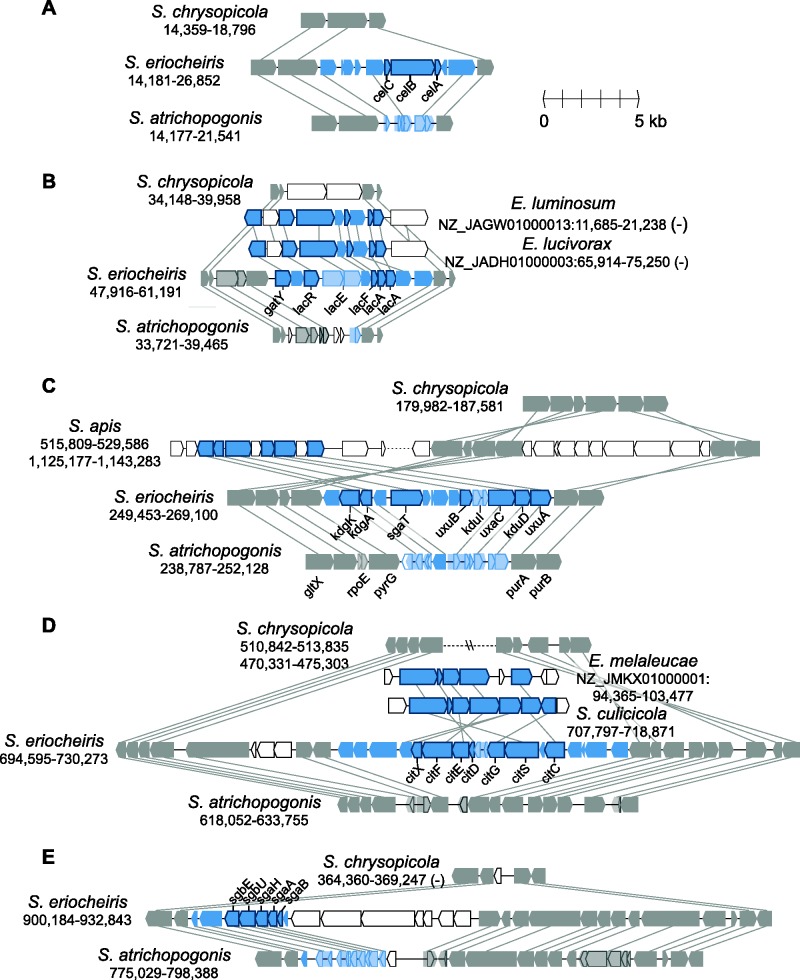
Gene organization of *S. eriocheiris*-specific regions. The panels correspond to the regions labeled in figure 2. The syntenic regions in other genomes are used to determine the boundaries of shared/unique regions. For draft genomes (i.e., *Entomoplasma *spp.), the contig accession numbers and start/end positions are labeled; for complete genomes (i.e., *Spiroplasma* spp.), only the start/end positions are labeled. Arrowheads represent genes in these regions drawn to scale. Conserved homologous genes are colored in gray and linked by gray lines. Genes that are putatively involved in horizontal transfers are highlighted in blue, the darker color ones indicate intact genes and the lighter color ones indicate pseudogenes (including arrowheads that show the remaining open reading frames). Genes without identifiable homologs in other genomes shown are represented by unfilled arrowheads.

Due to high levels of sequence divergence or the lack of homologs in other *Spiroplasma* lineages, using gene phylogenies to infer HGT is not feasible for most other candidates. For these, we examined the syntenic regions in other Mollicutes genomes listed in figure 1 based on the adjacent conserved genes (fig. 5; panels correspond to the regions labeled in fig. 2). In four out of the five cases (except for fig. 5*D*), these regions appeared to be present in the MRCA of the Mirum clade and absent in the sister Citri–Chrysopicola clade. However, the extensive pseudogenization that occurred in the *S. atrichopogonis* genome made most of these genes appear to be *S. eriocheiris*-specific in a simple three-way comparison of gene content (fig. 3).

Based on the gene phylogenies (fig. 4), the *S. eriocheiris*-specific set of cellobiose (fig. 5*A*) and ascorbate (fig. 5*E*) transporter genes may have been acquired from a Firmicutes donor. The gene cluster for lactose utilization shared similar gene organization in *Entomoplasma* (fig. 5*B*). However, due to several rearrangements, it was unclear whether these regions were acquired independently from different sources. Additionally, the gene losses observed in this region suggested that lactose is not an important sugar source for these *Spiroplasma* species, which is consistent with their arthropod-associated lifestyles. For the glucuronate metabolism gene cluster (fig. 5*C*), a similar region was found in *Spiroplasma apis* but not other available *Spiroplasma* genomes. Based on database searches, these glucuronate metabolism genes are most similar to those found in Bacillales, suggesting a Firmicutes origin. The region shown in figure 5*D* involved complex patterns because the synteny between Mirum and Chrysopicola clades was disrupted by rearrangements and whether the putative HGT region was present in *S. atrichopogonis* is unclear. Furthermore, although the gene cluster involved in citrate utilization was found in *Spiroplasma culicicola* and *Entomoplasma melaleucae*, gene order comparison and gene phylogenies suggested that these *S. eriocheiris* genes are more closely related to those found in peanut witches’-broom phytoplasma, which is an insect-transmitted phytopathogen also belonging to the class Mollicutes (Chung et al. 2013).

In addition to the regions shown in figure 5 that appeared to have been acquired as large clusters of functionally related genes, several other putative HGT events involved only one or few adjacent genes. These include genes for toxin A (with a glycosyltransferase domain), l-seryl-tRNA selenocysteine synthase (*selA*), 2-keto-3-deoxy-6-phosphogluconate aldolase (*kdgA*), and maltose transporter (*malX*). Intriguingly, in contrast to the larger scale HGT events illustrated in figure 5 where the more closely related Mollicutes or Firmicutes were inferred to be possible donors, these smaller scale HGT events appeared to involve the more distantly related Gammaproteobacteria based on BLASTP searches against GenBank.

### Gene Acquisition in *S. atrichopogonis* Mediated by MGEs

**Fig. 6.—:**
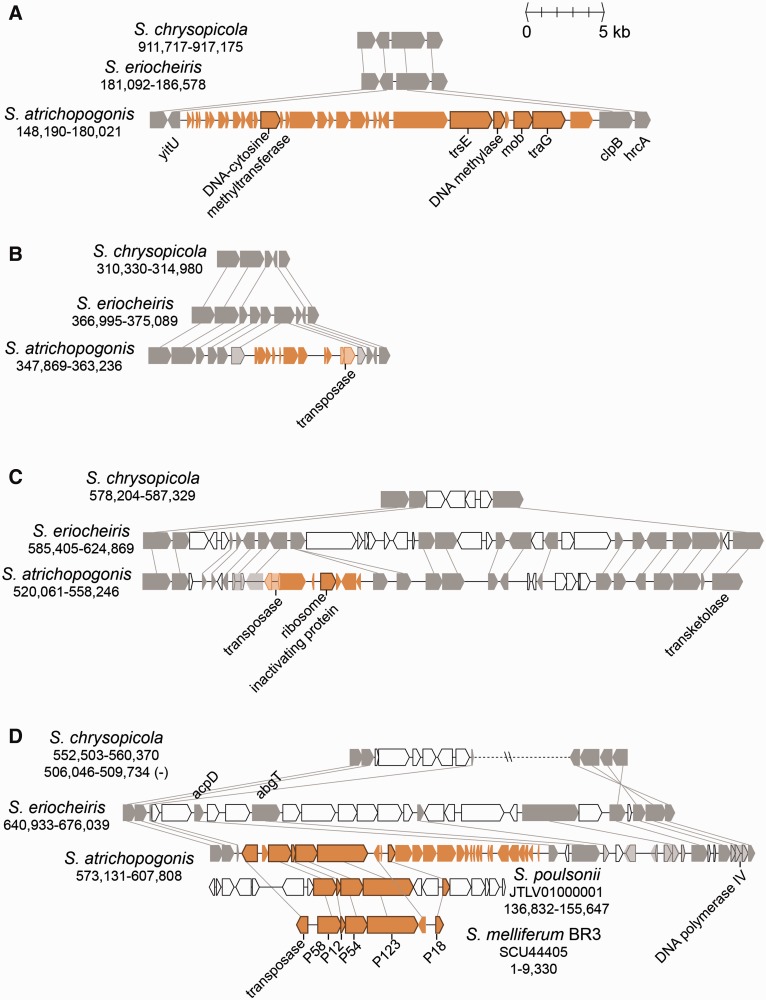
Gene organization of *S. atrichopogonis-*specific regions. The panels correspond to the regions labeled in figure 2. The color scheme is based on that used in figure 5, except for blue is replaced by orange for highlighting.

The genome alignment (fig. 2) showed that *S. atrichopogonis* has four major regions not found in the closely related *S. eriocheiris*. Examination of the gene content (fig. 6) suggested that these regions originated from insertions of MGEs. The region shown in figure 6*A* contains two conjugation-related genes (*trsE* and *traG*), as well as a putative plasmid recombination enzyme (*mob*), suggesting that this region is an integrative and conjugative element (ICE) (Burrus and Waldor 2004; Wozniak and Waldor 2010). Although the exact transfer mechanisms are unknown, ICEs have been identified as extrachromosomal DNA in *Spiroplasma citri* (Saillard et al. 2008) and present in the genome assembly of *Spiroplasma poulsonii* (Paredes et al. 2015). This *S. atrichopogonis* ICE also contains two DNA methylases, which may provide protection against host restriction endonucleases (Krüger and Bickle 1983). Intriguingly, the sequencing coverage of this region is approximately 50% of the genome average, suggesting that only half of the cells contained this ICE in the chromosome.

The regions shown in figure 6*B* and *C* both contain a pseudogenized transposase gene and a sequencing coverage similar to the genome average, indicating that these regions are remnants of stably integrated transposons. Similar to the ICE shown in figure 6*A*, these two putative transposons were also found in the genomes of *S. citri* (Carle et al. 2010) and *S. poulsonii* (Paredes et al. 2015), as well as in *S. mirum* (GenBank accession numbers CP002082 and CP006720), suggesting that these mobile DNA may facilitate the genetic exchanges among these *Spiroplasma* species. Notably, the transposon in figure 6*C* encodes a putative ribosome inactivating protein. The RIP domain (pfam00161) of this protein shares approximately 45% amino acid sequence similarity to those found in the verocytotoxin 1 produced by *Escherichia coli* and the Shiga toxin subunit A produced by *Shigella dysenteriae*. Because these enterobacterial toxins are implicated in the hemorrhagic colitis symptom (Karmali 1989), the presence of this putative toxin in *Spiroplasma* genome may partly explain the internal organ hemorrhages observed in *S. mirum*-infected mammals (Kirchhoff, Heitmann, et al. 1981; Kirchhoff, Kuwabara, et al. 1981).

The region highlighted in figure 6*D* corresponds to the region with an unexpected pattern of GC-skew in figure 2 (at ∼0.6 Mb), suggesting that this stretch of DNA has been inserted recently and has not been ameliorated yet (Lawrence and Ochman 1997). In addition to a transposase gene, this region encodes several adhesion-related proteins (ARPs; including P58, P12, P54, P123, and P18). The syntenic region could be found in several lineages belonging to the Citri clade. Previous functional characterization has shown that these ARPs are important in *S. citri* for its invasion into insect cells (Béven et al. 2012). Because these ARP genes are absent in *S. eriocheiris*, as well as in all available complete genomes from the Chrysopicola or Apis clade, it is reasonable to assume that *S. atrichopogonis* has acquired this region from a Citri-clade donor recently. The Illumina raw reads coverage of this region is approximately 3-fold of the genomic average. For the reads located at the junctions, approximately one-third supported the integration of this region in the chromosome, whereas the remaining two-thirds supported the region being a circular extrachromosomal element. This pattern suggested that this putative transposon might be still active in *S. atrichopogonis* and is consistent with the previous observations that these ARP genes may be located on the chromosome (Paredes et al. 2015) or extrachromosomal elements (Saillard et al. 2008).

## Discussion

### A Model for the Molecular Evolution Events in the Mirum Clade

**Fig. 7.—:**
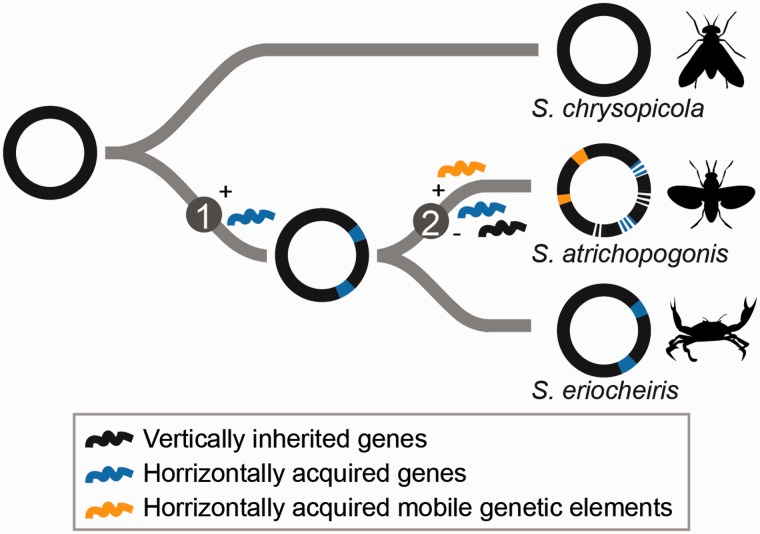
A model for the molecular evolution events in the Mirum clade. Circles represent the chromosomes; the hosts of extant species are drawn to the right. The differences in gene content between the Mirum and the Citri–Chrysopicola clades are best explained by acquisition of metabolic genes through HGT in the MRCA of the Mirum clade (step 1). Within the Mirum clade, the differences between species appeared to be results of MGE invasions and extensive pseudogenization (step 2) in the lineage leading to *S. atrichopogonis*.

Based on the results of our comparative and evolutionary analyses, we proposed a model for the genome evolution in the Mirum clade (fig. 7). Several HGT events from donors belonging to Tenericutes and Firmicutes (figs. 4 and 5) appeared to have occurred in the MRCA of the Mirum clade, which may have been facilitated by their coexistence in arthropod guts. Many of these horizontally acquired genes have been maintained in the lineage leading to *S. eriocheiris*, although it is unclear whether these genes contributed to its fitness or were associated with the host switch to aquatic crustaceans (fig. 3). On the other hand, the loss of DNA repair system, together with possible elevation in genetic drift (Kuo et al. 2009), led to the extensive pseudogenization as well as invasions of MGEs in *S. atrichopogonis*.

### Other Examples of Genome Degradation in Arthropod Symbionts

The pattern of genome degradation observed in *S. atrichopogonis* has been found in several other arthropod-associated bacteria (Moran et al. 2008; McCutcheon and Moran 2012). The most closely related example is *S. poulsonii*, which is a vertically transmitted reproductive parasite of *Drosophila* (Paredes et al. 2015). Other examples include *Borrelia recurrentis* in the phylum Spirochaetes (Lescot et al. 2008) and *Rickettsia prowazekii* belonging to Proteobacteria (Ogata et al. 2001). In these other examples, the species that experienced genome degradation all exhibit a higher level of virulence compared with the sister lineage that did not. However, in the case of *S. atrichopogonis*–*S. eriocheiris* comparison, the pattern is the opposite. It is unclear whether genome degradation and the increase in virulence are indeed correlated, and if so, the genetic explanation of this observation.

### Horizontally Transferred Genes: Source, Function, and Potential Link to Adaptation

For the HGT events occurred in the MRCA of the Mirum clade and involving multiple linked genes (fig. 5), the donors were most likely those sharing similar ecological niches (e.g., arthropod guts) and genomic characteristics (e.g., low GC content and the associated biased codon usage preference) with the recipient. Conceivably, these factors increased the probability for HGT to occur and the transferred genes to survive in the recipient genome. In terms of function, most of the putative horizontally acquired genes in *S. eriocheiris *encode transporters or metabolic enzymes (figs. 3 and 5). This observation is consistent with previous large-scale comparative analyses that new genes are more likely to be integrated at the periphery of genetic network and participate in limited protein–protein interactions (Lercher and Pál 2008; Cohen et al. 2011). Furthermore, Pál et al. (2005) suggested that the metabolic network evolution in *E. coli* was driven by adaptation to changing environments and the network grew mainly through acquiring genes involved in transport and catalysis of nutrients over the past 100 Ma. Thus, it is possible that at least some of the horizontally acquired genes have contributed to the adaptation of *S. eriocheiris*, such that these genes have been maintained in the *S. eriocheiris *genome while lost in the lineage leading to *S. atrichopogonis* (i.e., without the host switch). Because the switch to aquatic crustacean hosts has occurred at least two times within the genus *Spiroplasma* (fig. 1), it will be interesting to investigate the gene content evolution in *Spiroplasma penaei* (Nunan et al. 2005) and compare the findings with the *S. eriocheiris *inference reported here*.*

### Implications on Bacterial Species Description and Identification

Relating to the species concept for bacteria, two aspects concerning the limitation of bacterial strain characterization are reinforced by this study. Although the serological data indicated that *S. atrichopogonis* and *S. eriocheiris* are both distinct from *S. mirum *(Koerber et al. 2005; Wang et al. 2011), molecular phylogeny based on the 16S rRNA gene indicated that these three species are in fact quite closely related (fig. 1; *S. atrichopogonis* and *S. mirum* share 100% sequence identity, whereas *S. eriocheiris* is 99.7% identical to the previous two). In other taxonomic groups, such high levels of 16S rRNA sequence similarity would lead to the designation of these strains into the same species despite their differences in serotype (e.g., *E. coli* K12 and *E. coli* O157). Furthermore, there are several examples in *Spiroplasma* where new serotypes were designated at the time of novel species description, yet a “bridge” strain was later discovered to link multiple serogroups (Abalain-Colloc et al. 1993; Gasparich et al. 1993). On the other hand, similarity in serology or 16S rRNA gene does not necessarily translate to similarity in gene content or ecology. As highlighted in this and many other previous studies (Ochman et al. 2000; Gogarten et al. 2002; Gogarten and Townsend 2005; Lerat et al. 2005; Wiedenbeck and Cohan 2011), HGT could potentially change the gene content, pathogenicity, and ecology of a bacterial strain. For example, *Ureaplasma* is another genus in the class Mollicutes and contains two species of sexually transmitted pathogens that infect human urogenital tracts. A large-scale characterization of *Ureaplasma* clinical isolates demonstrated that recombination between serovars and extensive HGT has disrupted the association between serotypes and virulence, such that serotyping is of limited utility for diagnostic purpose (Xiao et al. 2011). Given these limitations, the initial characterization and the subsequent identification of Mollicutes strains remain a complex issue that requires integration of multiple approaches. The conventional emphasis on the use of serotyping and/or 16S rRNA gene sequencing may not be sufficient.

## Supplementary Material

Supplementary tables S1 and S2 are available at *Genome Biology and Evolution* online (http://www.gbe.oxfordjournals.org/).

Supplementary Data
