# Supplementary material for: Found and Lost: The Fates of Horizontally Acquired Genes in Arthropod-Symbiotic Spiroplasma
Source: Genome Biol Evol. 2015 Aug 8;7(9):2458–72. doi: 10.1093/gbe/evv160 (PMC4607517; doi:10.1093/gbe/evv160)
Supplement: Supplementary Data [file supp_7_9_2458__index.html]

Found and Lost: The Fates of Horizontally Acquired Genes in Arthropod-Symbiotic Spiroplasma — Supplementary Data 

# Found and Lost: The Fates of Horizontally Acquired Genes in Arthropod-Symbiotic *Spiroplasma*

## Supplementary Data

files

- Supplementary Data - docx file
- Supplementary Data - xls file
- Supplementary Data - xls file
