# Supplementary material for: Found and Lost: The Fates of Horizontally Acquired Genes in Arthropod-Symbiotic Spiroplasma
Source: Genome Biol Evol. 2015 Aug 8;7(9):2458–72. doi: 10.1093/gbe/evv160 (PMC4607517; doi:10.1093/gbe/evv160)
Supplement: Supplementary Data [file supp_evv160_New_Microsoft_Office_Word_Document.docx]

**Supplementary Materials**

**Supplementary Table S1.** Lists of GenBank accession numbers of the genes used for phylogenetic analyses shown in figs. 1 and 4.

**Supplementary Table S2.** Lists of homologous gene clusters among *S. atrichopogonis* (Sa), *S. eriocheiris* (Se), and the pan-genome of Citri-Chrysopicola (CC) clade strains listed in table 1.
